# External Morphology of *Lophiosilurus alexandri* Steindachner, 1876 during Early Stages of Development, and Its Implications for the Evolution of Pseudopimelodidae (Siluriformes)

**DOI:** 10.1371/journal.pone.0153123

**Published:** 2016-04-15

**Authors:** Fernando Massayuki Assega, José Luís Olivan Birindelli, Andréa Bialetzki, Oscar Akio Shibatta

**Affiliations:** 1 Programa de Pós-Graduação em Ciências Biológicas, Departamento de Biologia Animal e Vegetal, Centro de Ciências Biológicas, Universidade Estadual de Londrina, 86051–980, Londrina, PR, Brazil; 2 Programa de Pós-Graduação em Ecologia de Ambientes Aquáticos Continentais, Departamento de Biologia, Universidade Estadual de Maringá, Maringá, PR, Brazil; James Cook University, AUSTRALIA

## Abstract

Pseudopimelodidae are Neotropical catfishes characterized by having slightly to strongly depressed body in fully developed specimens. The largest species of the family with 500 mm SL, *Lophiosilurus alexandri*, experiences impressive changes in body shape during development, becoming extremely depressed when fully developed. Accordingly, *Lophiosilurus alexandri* is an ideal species to observe the morphological changes during ontogeny, and to seek solid interpretations on the polarity of characters. Specimens of distinct larval periods (yolk sac, flexion and postflexion; n = 186 specimens) and juvenile stages (n = 20) were analyzed. Changes in body shape, position of mouth and eye, morphology of fins and pigmentation were observed during the development of *Lophiosilurus*. Larvae (5.7–11.2 mm standard length) had pigmentation concentrated on the head and parts of body, eyes small and pigmented, short barbels, and well-developed finfold. Juveniles (15.9–28.1 mm standard length) had body shape similar to adult, with head depressed and bearing bony ridges, large mouth, dorsally-oriented eyes, small barbels and well-developed shoulder bulges (cleithral width). The greatest morphological changes in the development of *L*. *alexandri* occurred during the postflexion larval stage. Relative to standard length, measurements of snout length, head depth and body depth are smaller in juveniles than in larvae, but body width is larger. New interpretations on the phylogenetic characters related to these changes are provided in view of the two alternative hypotheses of the evolution of Pseudopimelodidae.

## Introduction

The search for the morphological changes that gave rise to the present diversity of forms and taxa continues to be one of the main goals of Evolutionary Biology. Many of these changes can in fact be interpreted as series of transformations of character states with genetic basis and functional expression. Nevertheless, the phylogenetic significance of these transformation series is unknown for most species of Neotropical fishes. One way to trace a transformation series is to study the ontogeny of a particular species [1]. The Biogenetic law, proposed by Haeckel [2] and reformulated by Nelson [1], says that the steps of an ontogenetic series can be directly translated into an ordered series of character states. That concept, however, has not been accepted among systematists [3]. The main arguments against Nelson’s Biogenetic law are that there is no evidence that certain body parts are more conservative or contain more information than other parts, and that the ontogenetic changes permit direct interpretations as ordered character states [4]. Besides these restrictions, Wayne [5] noted that the morphological diversity of adult forms of dogs and other domesticated animals depends on expressions during ontogeny. According to Lovejoy et al. [6], only a confident understanding of the phylogenetic relationships of a group can recover the polarization of characters.

The “pacamã”, *Lophiosilurus alexandri*, is a particularly interesting case for addressing this problem. *Lophiosilurus alexandri* is the largest species of the family Pseudopimelodidae, reaching 500 mm standard length [7] and 4 kg body mass [8]. This monotypic genus also exhibits the most highly modified morphology for the family. Its head is extremely depressed, wider than long, the eyes are dorsal, and the mouth is wide and prognathous [7]. In the phylogeny of Pseudopimelodidae proposed by Shibatta [9], *L*. *alexandri* is in a derived clade, sister to *Cephalosilurus*. Birindelli & Shibatta [10] corroborated the sister group relationship between *Lophiosilurus* and *Cephalosilurus*, but supported that clade sister to all other Pseudopimelodidae based on swim bladder musculature, a hypothesis that corroborated the findings of Ortega-Lara & Lehmann [11]. Therefore, given its large size, unique shape, and controversial phylogenetic position, *L*. *alexandri* is a potentially informative species for understanding morphological evolution in Pseudopimelodidae.

Endemic to the São Francisco River basin [12] in northeastern Brazil, *Lophiosilurus alexandri* is non-migratory and its reproduction involves the construction of a circular nest depression (40–50 cm diameter and 8–10 cm depth) in a shoal area. Spawning is parceled and the female lays large adhesive eggs (3.1 to 3.6 mm) in the center of the nest, which are then guarded and maintained by the male [13]. Upon hatching, the larvae remain at the bottom of the nest and do not move vertically into the water column. According to Tenório [12], it is among the most prized fishes in regional markets, but rarely captured in the upper São Francisco River basin. Due to its excellent flavor and the consistency of its flesh [8], the species has good potential for intensive aquaculture. Every year, juvenile *Lophiosilurus alexandri* are released into the São Francisco from a hatchery associated with UHE Três Marias as a compensatory measure for construction of the hydroelectric power plant.

Previous studies regarding the anatomy of *Lophiosilurus alexandri* included Nakatani *et al*. [14], who described its external morphology from egg to juvenile, and Guimarães-Cruz *et al*. [15], who described the internal anatomy of *L*. *alexandri* based on histological sections of the nervous and digestive systems. The present study contributes with details on the early development of the external morphology in *L*. *alexandri*, and new interpretations of phylogenetic characters previously employed by two alternative hypotheses on the evolution of Pseudopimelodidae.

## Material and Methods

Developmental series of *Lophiosilurus alexandri* were obtained from Estação de Piscicultura de Três Marias, CODEVASF, on the upper São Francisco River (Minas Gerais State). Individuals were reared from natural spawning of wild adults in flowing water. Specimens were collected between August 1994 and January 1996, fixed in 4% formaldehyde buffered with calcium carbonate and deposited in the fish collection of the Museu de Zoologia da Universidade Estadual de Londrina (MZUEL), Brazil.

The larval period was divided into three stages (yolk sac, flexion and postflexion) based on the degree of flexion of notochord and development of fins and their supporting elements using standards developed by Ahlstrom & Ball [16], and Kendall *et al*. [17] modified by Nakatani *et al*., [14]. Juveniles were determined by the complete development of fins and segmentation of fins rays [14]. A total of 206 specimens were analyzed, including 186 larvae from hatching to 42 days of development (24 yolk sac, 3 flexion and 159 postflexion larvae) and 20 juveniles (49 to 60 days) (Table 1).

**Table 1:** Minimum (Min), maximum (Max), average (Mean), standard deviation (SD) and coefficient of variance (CV) of relative body measurements in larvae and juveniles of *Lophiosilurus alexandr*i.

	Larval Period												Juvenile Period			
	Yolk sac (n = 24)				Flexion (n = 3)				Postflexion (n = 159)				Juvenile (n = 20)			
	Min—Max	Mean	SD	CV (%)	Min—Max	Mean	SD	CV (%)	Min—Max	Mean	SD	CV (%)	Min—Max	Mean	SD	CV (%)
1. SL (mm)	5.7–8.0	7.4	0.7	9.9	8.1–9.5	9.0	0.4	0	9.0–15.4	11.2	1.1	11.1	15.9–28.1	19.2	2.9	15.3
**Percentages of head length**																
9. ED	8.3–18.2	10.0	3.1	31.1	12.0–18.2	14.4	1.9	0	8.1–19.8	14.8	1.7	11.8	13.2–22.9	16.4	2.8	17.3
3. SnL	25.0–44.4	33.8	4.3	12.8	25.0–34.8	29.5	2.8	12.4	23.7–48.5	31.8	3.7	11.5	19.3–30.2	25.2	3.0	11.9
22. HD	40.0–111.1	82.8	16.5	19.9	53.3–82.6	67.8	7.9	0	32.3–87.0	61.8	8.7	14.0	28.6–47.7	35.9	5.2	14.4
19. IMBL	−	−	−	−	25.0–40.0	29.8	3.9	20.0	13.5–35.5	25.4	4.0	15.6	16.5–43.3	24.6	7.5	30.6
18. OMBL	−	−	−	−	20.0–52.2	41.6	8.9	17.3	29.3–58.8	40.8	5.7	13.8	19.7–42.5	31.7	7.3	23.0
17. MXBL	−	−	−	−	40.0–75.0	49.6	9.2	7.5	44.2–96.8	65.4	7.2	14.2	43.1–59.3	50.3	5.4	10.8
**Percentages of standard length**																
2. HL	0.5–1.5	1.0	0.2	17.5	18.1–27.3	24.2	2.7	1.4	23.7–39.8	32.5	3.0	11.7	32.2–39.5	36.0	2.1	5.8
23. BD	2.2–2.8	2.4	0.2	10.8	24.7–29.6	27.9	1.5	3.4	18.1–28.9	22.8	1.9	9.7	15.6–22.4	18.5	1.7	9.1
8. CPD	−	−	−	−	−	−	−	−	6.0–11.1	8.1	0.9	11.3	7.6–11.3	9.2	0.9	10.2
15. CPL	−	−	−	−	−	−	−	−	11.7–20.7	16.4	2.0	12.2	10.3–20.8	15.8	2.7	16.9
14. PSL	−	−	−	−	−	−	−	−	6.7–18.2	13.7	2.3	16.8	9.6–19.5	15.4	2.1	13.8
10. PVRL	−	−	−	−	−	−	−	−	6.3–17.2	11.3	2.2	19.7	10.7–18.8	13.0	1.9	14.8
12. DSL	−	−	−	−	−	−	−	−	5.4–16.8	10.4	2.0	19.5	9.1–15.6	11.9	1.7	14.3
11. DFBL	−	−	−	−	−	−	−	−	10.8–18.2	14.0	1.5	10.7	10.7–19.4	14.6	2.1	14.3
13. AFBL	−	−	−	−	−	−	−	−	12.5–19.2	15.4	1.3	8.2	11.8–17.1	14.1	1.6	11.2
7. PAL	−	−	−	−	−	−	−	−	62.0–75.3	67.1	2.5	3.7	65.6–74.3	70.1	2.5	3.6
16. IW	−	−	−	−	−	−	−	−	24.8–45.5	35.8	3.7	10.3	27.2–42.5	34.7	4.5	12.9
20. BW	−	−	−	−	−	−	−	−	19.7–32.0	25.5	2.8	19.5	27.0–33.8	30.7	1.7	9.6
21. IPW	−	−	−	−	−	−	−	−	6.1–14.6	8.4	1.6	11.1	10.7–15.9	13.6	1.3	5.6
4. PPL	−	−	−	−	−	−	−	−	24.1–33.6	19.1	1.6	5.4	21.3–35.0	29.1	3.4	11.6
6. PVL	−	−	−	−	−	−	−	−	38.3–51.4	45.6	2.3	5.1	38.4–53.7	48.0	3.2	6.7
5. PDL	−	−	−	−	−	−	−	−	32.4–52.9	37.6	2.3	6.1	33.8–43.5	38.4	2.4	6.3

AFBL = anal-fin base length; BD = body depth; BW = body width; CPD = caudal peduncle depth; CPL = caudal peduncle length; DFBL = dorsal-fin base length; DSL = dorsal-fin spine length; ED = eye diameter; OMBL = outer mentonian barbel length; HD = head depth; HL = head length; IMBL = inner mentonian barbel length; IPW = interpelvic width; IW = interorbital width; MXBL = maxillary barbel length; n = number of individuals analyzed; PAL = Preanal length; PDL = predorsal length; PPL = prepectoral length; PSL = pectoral-fin spine length; PVL = prepelvic length; PVRL = Pelvic-fin ray length; SL = standard length; SnL = snout length; − = not measured.

For each stage of development, specimen illustrations were made with the aid of a camera lucida coupled to a stereomicroscope. Multiple illustrations were made for stages that exhibited sudden changes in morphology. Twenty-three measurements were obtained on juveniles using a digital caliper accurate to 0.01 mm, and on larvae using an ocular micrometer attached to a stereoscopic microscope: anal-fin base length (AFBL), body depth (BD), body width (BW), caudal peduncle depth (CPD), caudal peduncle length (CPL), dorsal-fin base length (DFBL), dorsal-fin spine length (DSL), eye diameter (ED), head depth (HD), head length (HL), inner mentonian barbel length (IMBL), interorbital width (IW), interpelvic width (IPW), maxillary barbel length (MXBL), outer mentonian barbel length (OMBL), preanal length (PAL), predorsal length (PDL), prepectoral length (PPL), pectoral-fin spine length (PSL), prepelvic length (PVL), pelvic-fin ray length (PVRL), standard length (SL), and snout length (SnL),

Morphometric variables were expressed as percentages of standard length, except for subunits of the head, expressed as percentages of head length. Measurements of width and depth of body and head were taken on juveniles and adults of other species of Pseudopimelodidae (see list of comparative material) for comparison. Meristic data included the number of rays of the pectoral, pelvic, dorsal and anal fins, and total preanal and postanal myomeres. Correlation analyses were carried out using Microsoft Excel, and box plot analysis using Past v. 2.11 [18]. Institutional abbreviations follow Sabaj Pérez [19].

### Material Examined

*Lophiosilurus alexandri*: MZUEL 5681, 3, 7.6 to 8.0 mm SL, Estação de Piscicultura de Três Marias, CODEVASF, on the upper São Francisco River, Minas Gerais State; MZUEL 5682, 37, 8.7 to 9.5 mm SL, same as MZUEL 5681; MZUEL 5683, 21, 9.7 to 10.4 mm SL, same as MZUEL 5681; MZUEL5684, 23, 10.8 to 11.3 mm SL, same as MZUEL 5681; MZUEL 5685, 20, 11.0 to 11.7 mm SL, same as MZUEL 5681; MZUEL 5686, 25, 10.6 to 11.7 mm SL, same as MZUEL 5681; MZUEL 5687, 19, 10.7 to 11.7 mm SL, same as MZUEL 5681; MZUEL 5688, 13, 11.4 to 12.3 mm SL, same as MZUEL 5681; MZUEL 5689, 6, 13.3 to 15.2 mm SL, same as MZUEL 5681; MZUEL 5690, 7, 14.4 to 15.4 mm SL, same as MZUEL 5681; MZUEL 5691, 7, 16.9 to 18.3 mm SL, same as MZUEL 5681; MZUEL 5692, 8, 15.9 to 21.2 mm SL, same as MZUEL 5681; MZUEL 5693, 6, 18.4 to 20.0 mm SL, same as MZUEL 5681; MZUEL 5694, 3, 25.1 to 28.1 mm SL, same as MZUEL 5681; MZUEL 5702, 3, 7.4 to 7.5 mm SL, same as MZUEL 5681; MZUEL 5703, 3, 8.1 to 8.3 mm SL, same as MZUEL 5681; MZUEL 5704, 3, 8.6 to 9.2 mm SL, same as MZUEL 5681; MZUEL 5705, 3, 9.0 to 9.7 mm SL, same as MZUEL 5681; MZUEL 5706, 3, 10.1 to 10.2 mm SL, same as MZUEL 5681; MZUEL 5707, 3, 9.6 to 10.3 mm SL, same as MZUEL 5681; MZUEL 5708, 3, 10.0 to 10.7 mm SL, same as MZUEL 5681; MZUEL 5709, 3, 10.6 to 11.0 mm SL, same as MZUEL 5681; MZUEL 5710, 3, 10.6 to 10.8 mm SL, same as MZUEL 5681; MZUEL 5711, 3, 10.6 to 10.8 mm SL, same as MZUEL 5681; MZUEL 5712, 3, 10.4 to 11.2 mm SL, same as MZUEL 5681; MZUEL 5713, 3, 10.5 to 11.0 mm SL, same as MZUEL 5681; MZUEL 5714, 3, 10.5 to 10.8 mm SL, same as MZUEL 5681; MZUEL 5715, 2, 10.5 to 11.2 mm SL, same as MZUEL 5681; MZUEL 5716, 3, 10.5 to 11.2 mm SL, same as MZUEL 5681; MZUEL 5717, 3, 10.7 to 11. 1 mm SL, same as MZUEL 5681; MZUEL 5718, 3, 10.6 to 11.0 mm SL, same as MZUEL 5681; MZUEL 5719, 3, 11.0 to 11.3 mm SL, same as MZUEL 5681; MZUEL 5720, 3, 10.4 to 10.8 mm SL, same as MZUEL 5681; MZUEL5721, 2, 12.5 to 12.7 mm SL, same as MZUEL 5681; MZUEL 5722, 1, 11.6 mm SL, same as MZUEL 5681; MZUEL 5724, 3, 5.7 to 6.5 mm SL, same as MZUEL 5681; MZUEL 5725, 3, 6.0 to 6.6 mm SL, same as MZUEL 5681; MZUEL 14133, 1, 30.8 mm SL, rio São Francisco, represa de Três Marias, Minas Gerais State; MZUEL 14134, 1, 28.3 mm SL, same as MZUEL 14133; MZUEL 14135, 1, 29.6 mm SL, same as MZUEL 14133; MZUEL 14136, 1, 33.2 mm SL, same as MZUEL 14133; MZUEL 14137, 1, 24.9 mm SL, same as MZUEL 14133; MZUEL 14138, 1, 33.2 mm SL, same as MZUEL 14133; MZUEL 14139, 1, 36.6 mm SL, same as MZUEL 14133; MZUEL 14140, 1, 39.1 mm SL, same as MZUEL 14133; MZUEL 14141, 1, 38.5 mm SL, same as MZUEL 14133; MZUEL 14142, 1, 41.4 mm SL, same as MZUEL 14133; MZUEL 14143, 1, 33.9 mm SL, same as MZUEL 14133.

### Comparative material

*Batrochoglanis acanthochiroides*: USNM 121270, holotype, 106.0 mm SL, lake Maracaibo basin, río San Juan, tributary of río Motatán; USNM 121276, 2 paratypes, 41.1 to 53.2 mm SL, lake Maracaibo basin, río Machango; USNM 121278, 1 paratype, 27.2 mm SL, río Jimeles, tributary of río Motatán; USNM 121279, 2 paratypes, 33.0 to 73.6 mm SL, río Tachira, río Catatumbo system; USNM 121271, 4 paratypes, 33.6 to 54.1 mm SL, río San Pedro, río Motatán system; USNM 121273, 2 paratypes, 51.0 to 80.4 mm SL, río San Juan, tributary of río Motatán. *Batrochoglanis raninus*: AMNH 78053, 4, 33.7 to 42.6 mm SL, río Tahuayo, tributary of río Amazonas, Huasi village; INPA 7343, 1, unmeasured, rio Tocantins, Base 4; INPA 8057, 1, 67.1 mm SL, rio Tocantins, igarapé Pucuruizinho, Tucuruí; INPA 9522, 1, 33.2 mm SL, rio Guaporé, 5 km upstream Costa Marques; MNHN A-9942, syntype, 70.4 mm SL, rio La Mana; MZUSP 6333, 1, 50.0 mm SL, lago Castro, rio Purus mouth; MZUSP 22126, 7, 27.8 to 38.5 mm SL, rio Utinga, Belém; MZUSP 22263, 1, 31.2 mm SL, Paissandu, Oriximiná; MZUSP 23023, 1, 33.2 mm SL, rio Arani, Arari waterfall, Ilha de Marajó; MZUSP 23029, 1, 39.2 mm SL, igarapé Apéu, Boa Vista, Castanhal; MZUSP 23376, 8, 49.2 to 75.0 mm SL, igarapé Manduaçu, paraná de IUPIÁ, NW of Fonte Boa; MZUSP 23407, 24, 21.7 to 74.4 mm SL, igarapé Tucuxi, Ati-Paraná, NW of Fonte Boa; MZUSP 23659, 13, 16.5 to 29.4 mm SL, igarapé Jaramacaru, tributary of Cuminá; MZUSP 24030, 5, unmeasured, rio Tocantins, igarapé Mapará, paraná Samuuna. *Batrochoglanis villosus*: AMNH 04419, 1, 88.6 mm SL, Potaro river, Tumatumari; AMNH 13658, 1, 102.4 mm SL, Essequibo river, Rockstone; AMNH 55370, 4, 25.8 to 78,0 mm SL, opposite creek to camp ca. 2 km downstream of Cow falls, Nickerie; ANSP 135756, 1, 50.6 mm SL, río Urbana (Urbani) in Maripa-Las Trincheras road, 7°18’N 65°00’W; ANSP 135903, 1, 91.4 mm SL, río Caura at Jabillal, 6°57’N 64°50’W; ANSP 139883, 1, 74.3 mm SL, small tributary of río Mato (left bank), 7°08’N 65°10’W; ANSP 160315, 1, 61.3 mm SL, small tributary of río Caura near confluence with río Orinoco, 7°37’48”N 64°50’42”W; FMNH 53219, holotype, 118.0 mm SL, Potaro Landing; FMNH 53220, paratype, 61.8 mm SL; FMNH 53571, paratype, 38.0 mm SL; INPA 3088, 1, 163.2 mm SL, rio Uatumã, Cachoeira Morena; INPA 4188, 1, 77.5 mm SL, rio Xingu, Babaquara island; INPA 5719, 1, 98.6 mm SL, rio Trombetas, upstream of Cachoeira Porteira; INPA 6589, 1, 119.2 mm SL, rio Marauiá, tributary of rio Negro; INPA 6800, 2, 41.0 to 42.6 mm SL, rio Jamanxim, tributary of rio Tapajós, Terra Preta island; INPA 6994, 3, 59.6 to 80.6 mm SL, rio Tapajós, at Pimental; INPA 7085, 1, 117.2 mm SL, rio Tapajós, at Pimental; INPA 9520, 1, 180.8 mm SL, rio Tocantins, Tucurui market; INPA 9518, 2, 86.8 to 97.4 mm SL, igarapé Jatuarana, approximately 2 km above Samuel hydroelectric power plant; MZUSP 6645, 1, 88.2 mm SL, igarapé of lago Manacapuru; MZUSP 7109, 8, 49.8 to 90.8 mm SL, igarapé in the left margin of rio Canumã, Canumã; MZUSP 7296, 2, 61.1 to 61.65 mm SL, igarapé of rio Marau, Maués; MZUSP 7356, 26, 44.4 to 110.1 mm SL, igarapé Limãozinho, Maués; MZUSP 7416, 1, 93.4 mm SL, igarapé of lago Saracá, at Silves; MZUSP 7445, 2, 52.8 to 85.1 mm SL, rio Sanabani, at Silves; MZUSP 7487, 1, 52.2 mm SL, igarapé tributary of Sanabani, at Silves; MZUSP 8497, 1, 51.0 mm SL, igarapé tributary of left margin of Mapiri, Santarém; MZUSP 8414, 1, 58.3 mm SL, igarapé Jacundá, Alter do Chão, Santarém; MZUSP 21840, 1, 108.6 mm SL, rio Tapajós, Cachoeira Lombo de Anta, near São Luiz; MZUSP 23042, 3, 46.4 to 66.8 mm SL, lago Jacaré, rio Trombetas; MZUSP 23293, 1, 181.8 mm SL, lagoa Central, left margin of rio Negro between rio Camaraú and rio Apaú; MZUSP 23301, 1, 35.6 mm SL, rio Jauperi, beach at 30 km above mouth; MZUSP 23548, 2, 58.7 to 77.0 mm SL, igarapé Açu, 7 km below Santo Antônio do Içá, left margin of rio Solimões; MZUSP 24061, 1, 39.0 mm SL, rio Tocantins, igarapé Limão, Baião; MZUSP 24286, 1, 63.8 mm SL, rio Tapajós, São Luiz; MZUSP 24903, 1, 178.1 mm SL, lago Janauacá and vicinity, rio Solimões; MZUSP 30820, 6, 34.8 to 89.0 mm SL, rio Tefé, Ipanema de Baixa; MZUSP 30821, 1, unmeasured, Rio Branco, Cachoeira do Bem-querer; MZUSP 30822, 7, 38.2 to 78.6 mm SL, rio Amapá, Cachoeira Grande; MZUSP 30823, 9, 31.2 to 48,6 mm SL, rio Tapajós, Pederneiras, below Itaituba; MZUSP 30824, 3, unmeasured, Rio Branco, Cachoeira do Bem-querer; MZUSP 30825, 1, 44.6 mm SL, rio Amapá, Cachoeira Grande; MZUSP 30826, 1, 37.4 mm SL, rio Tapajós, São Luiz above Itaituba; MZUSP 30836, 1, 138.8 mm SL, rio Tapajós, Alter do Chão, north beach; MZUSP 30835, 2, 79.4 to 158.6 mm SL, rio Urariqüera, Maracá island; MZUSP 31092, 1, 157.2 mm SL, rio Negro, São Gabriel da Cachoeira; MZUSP 31095, 1, 145.1 mm SL, rio Negro, Cachoeira de São Gabriel; MZUSP 31097, 1, 194.2 mm SL, upper rio Negro, confluence to igarapé do Igará, São Pedro; MZUSP 37770, 8, 30.9 to 108.7 mm SL, igarapé Tarumã, 10 km above barra do Canumã, Aripuanã; MZUSP 37786, 6, 36.3 to 107.2 mm SL, igarapé do Poraquê, below Dardanelos, Humboldt, Aripuanã; MZUSP 37811, 4, unmeasured, igarapé Ingazeiro, 20 km above boca do Canumã, rio Aripuanã, below Dardanelos, Aripuanã. *Cephalosilurus fowleri*: ANSP 172158, 2, 39.2 to 199.5 mm SL, rio Tatu, at barra do Cocos, 7 km S of Cocos, rio São Francisco basin; FMNH 54254, holotype, 301.4 mm SL, rio São Francisco, Barra; MCP 14094, 1, 328.8 mm SL, rio São Francisco, Três Marias and Pirapora; MCP 14126, 1, 262.6 mm SL, same as MCP 14094; MCP 16675, 2, 44.9 to 128.0 mm SL, same as ANSP 172158; MZUSP 38097, 1, 244.6 mm SL, rio Paracatu; MZUSP 24647, 1, 277.0 mm SL, same as MZUSP 38097. *Cephalosilurus albomarginatus*: FMNH 53221, holotype, 75 mm SL, Tukeit; FMNH 53222, 8 paratypes, 21.5 to 58.5 mm SL, downstream rio Potaro; FMNH 53572, 1 paratype, 68.8 mm SL, Waratuk; ROM 61336, 27, 29.0 to 88.4 mm SL, Tukeit falls, Potaro river, Essequibo basin; ROM 61482, 2, 50.5 to 78.8 mm SL, creek about 1 km below Tukeit falls at SE side of a tributary of Potaro river, Essequibo basin. *Cephalosilurus apurensis*: MBUCV-V-15379, 1, 165.2 mm SL, río Apure, in front of Apurito island, Apure. *Cruciglanis pacifici*: IMCN 113, 1, 91.6 mm SL, río Dagua basin, río San Cipriano at San Cipriano village, Buenaventura. *Pseudopimelodus bufonius*: INPA 1979, 1, 32.4 mm SL, rio Urariquera, Ilha de Maracá, first waterfall downstream island; INPA 4884, 1, 203.4 mm SL, rio Urariquera, Ilha de Maracá, furo Santa Rosa; INPA 6202, 1, 139.0 mm SL, rio Trombetas, Cachoeira Vira-Mundo; INPA 8058, 1, 68.8 mm SL, rio Uatumã, Balbina, immediately below dam; INPA 9514, 4, 56.3 to 87.9 mm SL, rio Mucajai; INPA 9516, 7, 66.4 to 95.6 mm SL, rio Jamari, diversion channel of Samuel hydroelectric power plant. *Pseudopimelodus charus*: ANSP 172156, 1, 53.4 mm SL, rio Tatu (Bahia do Coco), tributary of rio Itaguari/rio Carinhanha, 14°14’22”S 44°31’42”W; ANSP 172157, 2, 70.0 to 71.7 mm SL, rio Verde Grande, along road from Montes Claros to Janaúba, 16°39’01”S 43°42’49”W; MZUSP 39278, 1, 93.4 mm SL, rio São Francisco, Ilha Grande; MZUSP 39752, 3, 121.2 to 123.4 mm SL, rio São Francisco, Formoso hidroelectric power plant area; MZUEL 14144, 1, 171.3 mm SL, rio Pardo Grande mouth, tributary of rio das Velhas, Conselheiro da Mata. *Pseudopimelodus mangurus*: MCP 44779, 2, 123.5 to 134.7 mm SL, rio Uruguai, Monte Caseros; MCP 10336, 1, 129.0 mm SL, rio Uruguai, near mouth of arroio Cacaréu, Uruguaiana; MCP 12685, 1, 146.1 mm SL, rio Uruguai, Porto Santo Izidro, São Nicolau; MCP 13087, 1, 173.7 mm SL, rio Uruguai, Porto Santo Izidro, São Nicolau. *Pseudopimelodus pulcher*: AMNH 40127, 5, 37.5 to 62.4 mm SL, río Chapare, Villa Tunari. *Pseudopimelodus schultzi*: USNM 121258, 1, 150.3 mm SL, río Magdalena; USNM 175310, 1, 99.9 mm SL, río Magdalena, río Viego. *Microglanis iheringi*: USNM 121985, paratype, 1, 31.3 mm SL, río Turmero, Portuguesa; CAS 64403, 3, 27.4 to 41.0 mm SL, río Orinoco. *Microglanis parahybae*: MNRJ 15989, 5, 30.3 to 34.2 mm SL, rio Dois Rios, rio Paraíba do Sul basin; MNRJ 16047, 5, 28.6 to 38.9 mm SL, rio Muriaé, rio Paraíba do Sul basin. *Microglanis poecilus*. INPA 28575, 3, 18.6 to 20.6 mm SL, rio Aripuanã, rio Madeira basin; INPA 28576, 3, 19.8 to 20.4 mm SL, igarapé Ano Bom, rio Branco basin; INPA 8052, 3, 24.8 to 26.2 mm SL, igarapé Maracá, rio Branco basin; INPA 6828, 3, 19.2 to 25.8 mm SL, rio Jamanxin, rio Tapajós basin, 5°27’11”S 55°52’40”W; ROM 60738, 1, 22.5 mm SL, unknown trib Essequibo river, 4°46’20”N 58°45’W; ROM 62390, 1, 17.1 mm SL, Shimiri Stream, Yawiri, Essequibo basin, 4°42’13”N 58°42’43”W; ROM 62391, 1, 17,1 mm SL, Essequibo, 4°48’22”N 58°46’14”W.

## Results

### Morphological descriptions

#### Yolk sac (n = 24)

From hatching to day 2 of development. Standard length 5.7 to 8.0 mm (Table 1). Total number of visible myomeres 38 to 43 (14 to 17 preanal and 23 to 26 postanal).

**Fig 1:**
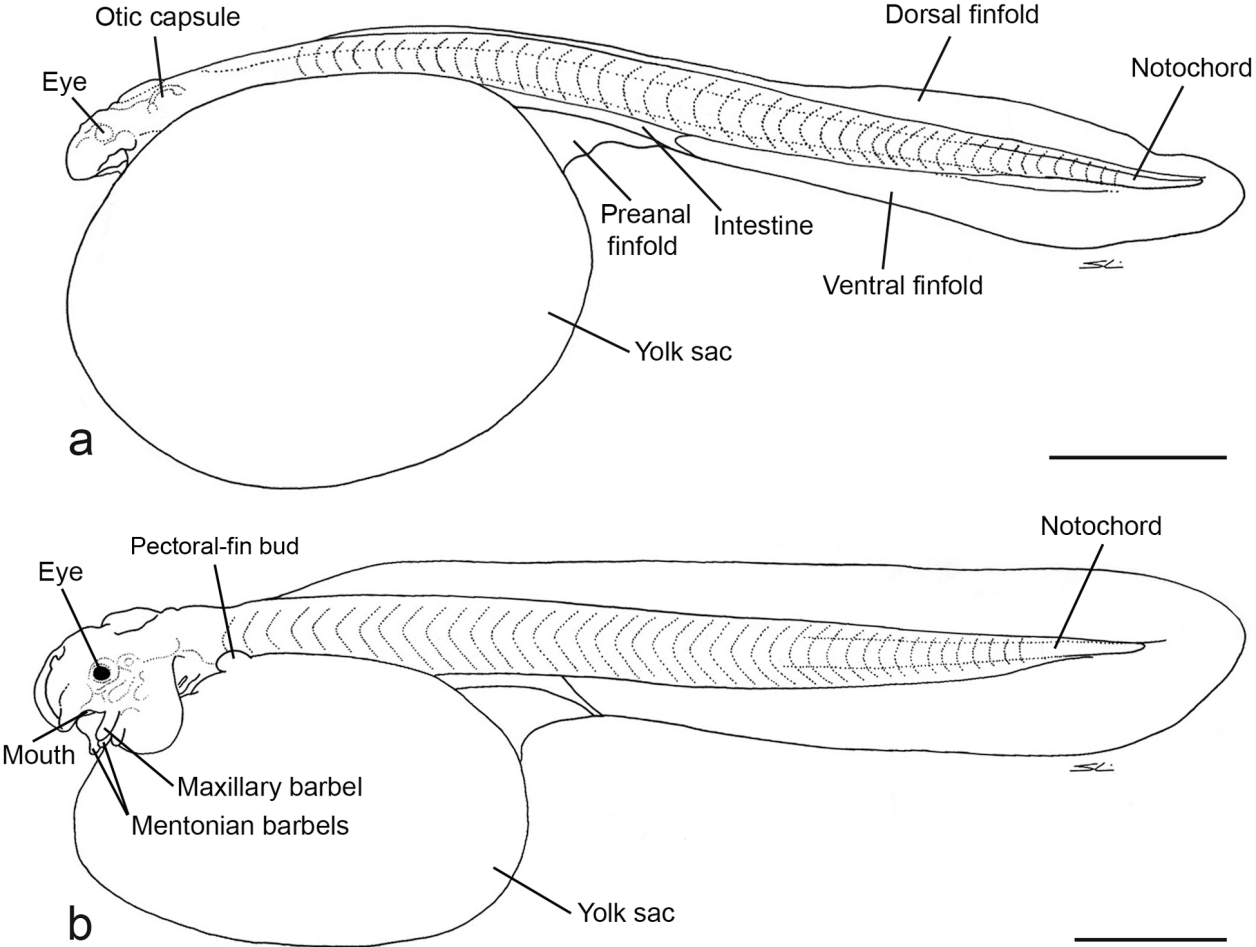
Lateral view of larvae of *Lophiosilurus alexandri*: a) newly hatched, MZUEL 5725; b) one-day old, MZUEL 5681. Scale bar = 1 mm.

Body elongate, transparent, without dark pigmentation. Yolk sac very large immediately after hatching (Fig 1a), decreasing in volume in specimens one or two days post-hatching (Fig 1b). Notochord straight, visible as transparency for entire length.

Eyes small, not pigmented in newly hatched specimens, but pigmented in one day old specimens. Nostrils undeveloped. Small gill opening present within the first day.

Mouth not formed soon after hatching; visible in subterminal position after the first day of development. Buds of maxillary and mentonian barbels present only within the 1^st^ day of development. From the second day of development, barbels present. Otic capsule with one semicircular canal visible, and two otholits. Intestine short, anus closed. Primordial fin (finfold) well developed, hyaline, circling the body from the post-cephalic region to the post yolk sac region.

#### Preflexion

Stage absent in *L*. *alexandri* since after termination of the yolk sac stage hypurals present.

#### Flexion (n = 3)

Stage observed on day 3 of development; standard length 8.1 to 8.3 mm. Total number of myomeres 40 (15 preanal and 25 postanal).

Head deep, longer than wide, rounded shape in lateral view. Mouth opened but incompletely developed. Maxillary and mentonian barbels distinct and short. Eyes pigmented. Nostril, gill opening and axillary gland not formed. Intestine small, not reaching the middle region of body; anus closed. Notochord visible by transparency with flexion in caudal region.

Primordial dorsal, pectoral, anal and caudal fins with incipient rays. Primordial dorsal fin anterior to middle of body, confluent with dorsal finfold; pectoral-fin bud small, rounded, when adpressed, slightly surpassing vertical through dorsal-fin origin. Primordial anal fin broadly rounded, visible as thick membrane included within in ventral finfold. Incipient caudal fin wing shaped, visible within confluent portions of dorsal and ventral finfolds; hypurals forming as cartilage. Head and body slightly pigmented with dendritic chromatophores containing dark pigment. Large yolk sac persistent, unpigmented.

#### Postflexion (n = 159)

Stage observed four to 42 days after hatching. Standard length 8.6 to 15.4 mm. Total myomeres 38 to 41 (14 to 15 preanal and 24 to 26 postanal). Longest stage of larval development with a variety of morphological changes.

**Fig 2:**
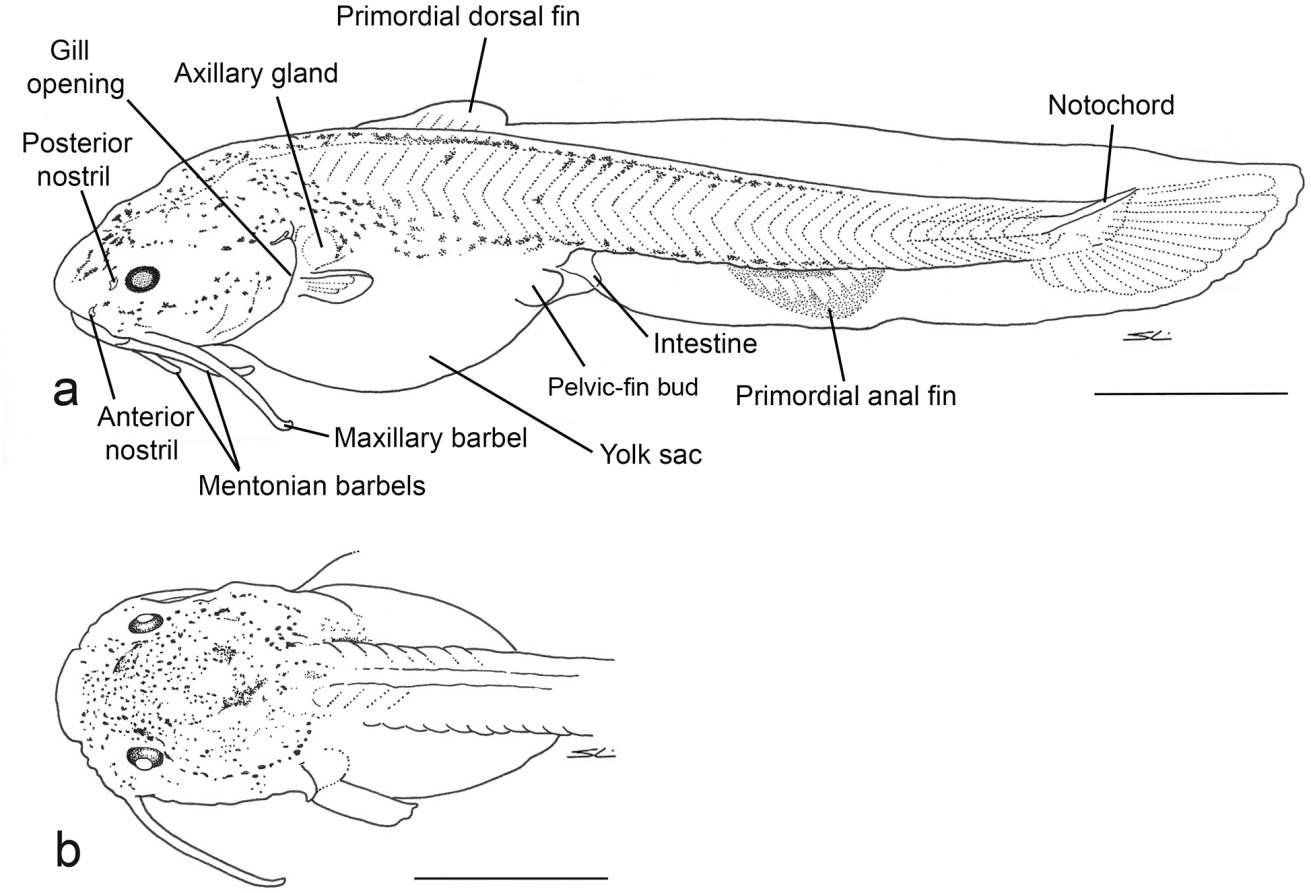
*Lophiosilurus alexandri* four days after hatching, MZUEL 5682, in the early stage of postflexion: a) lateral view; b) dorsal view of head. Scale bar = 1 mm.

**Fig 3:**
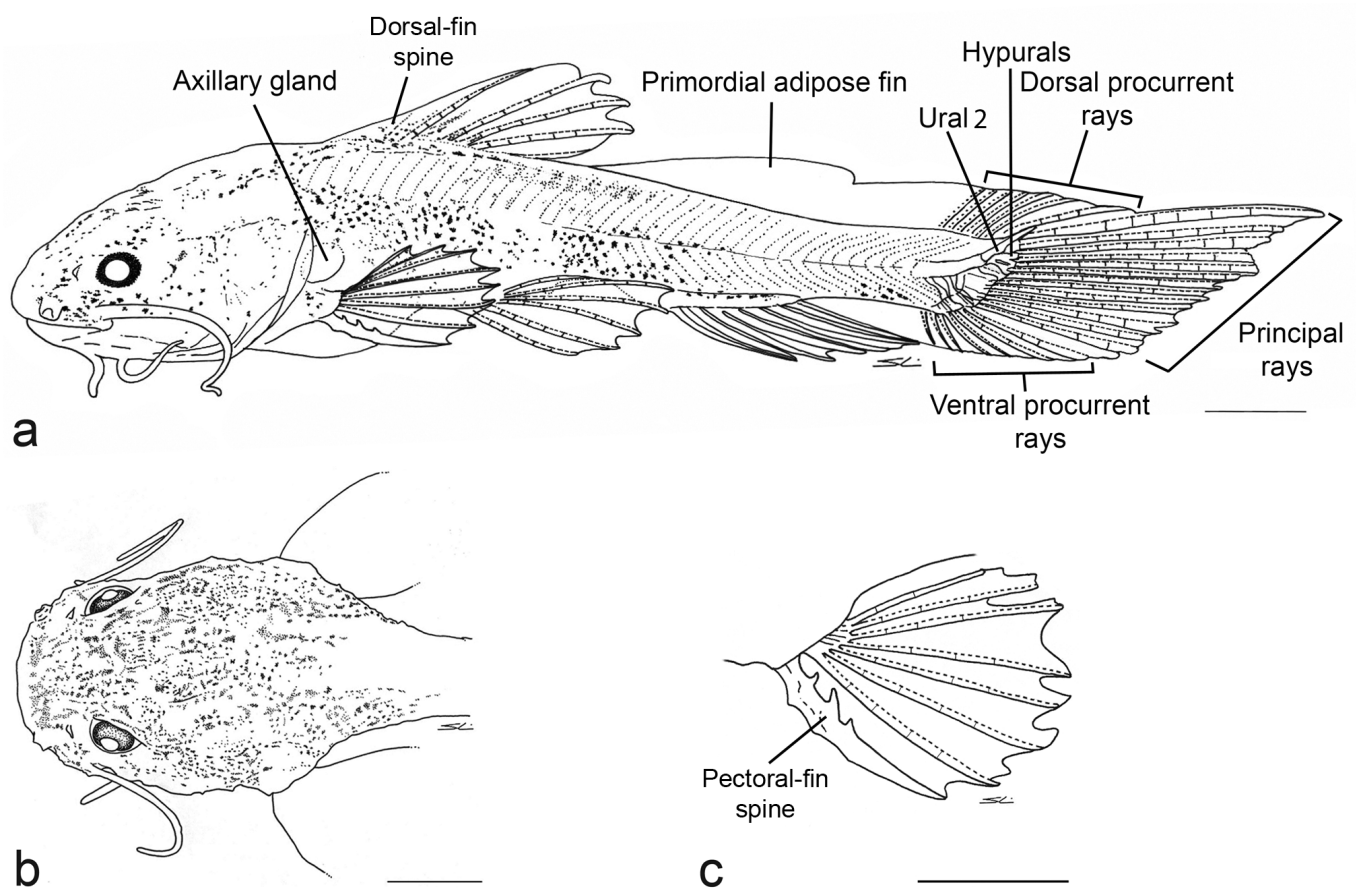
*Lophiosilurus alexandri* 17 days after hatching, MZUEL 5686, early mid-stage of postflexion: a) lateral view; b) dorsal view of anterior region; c) left pectoral fin in dorsal view. Scale bar = 1 mm.

**Fig 4:**
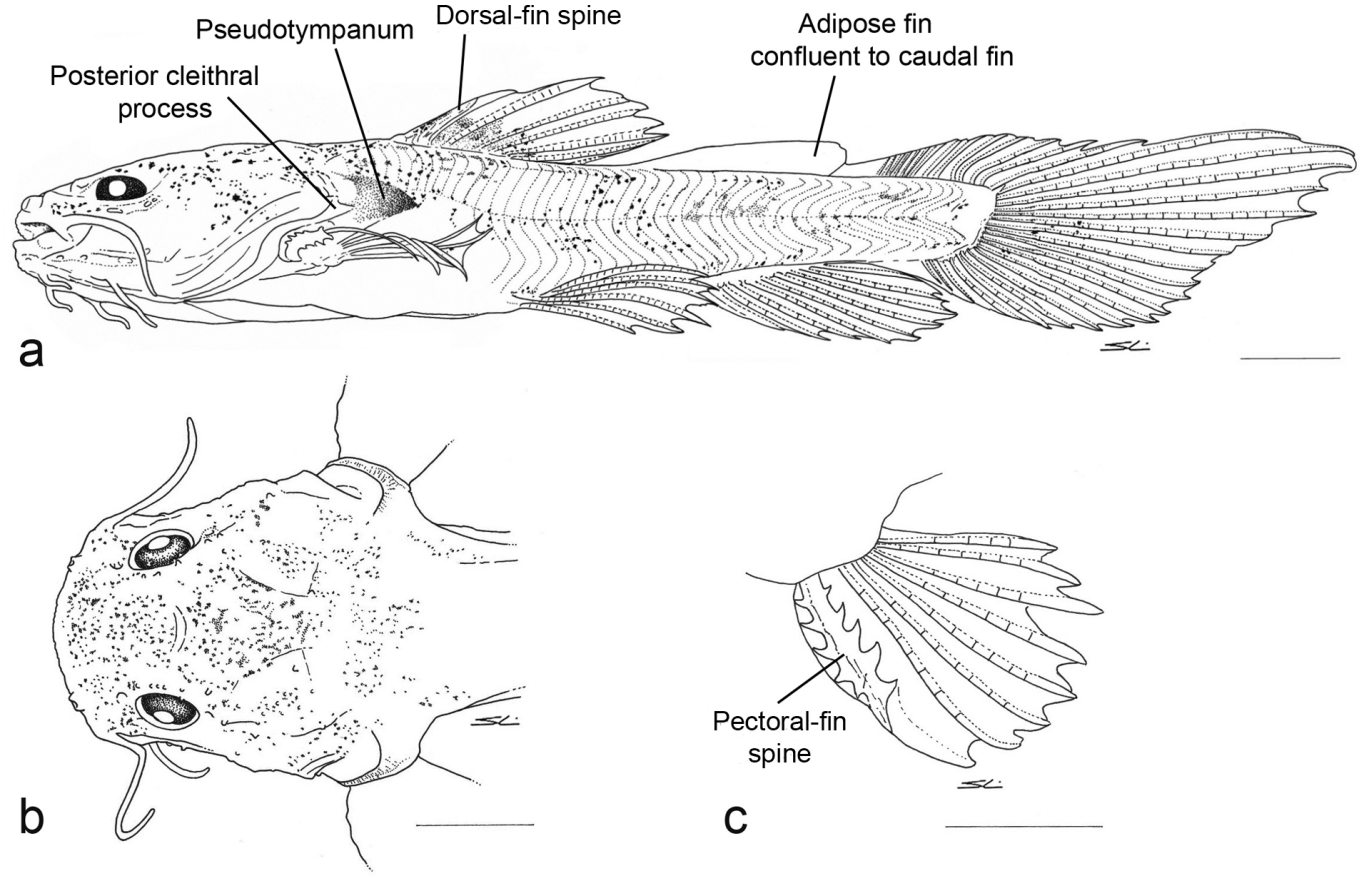
*Lophiosilurus alexandri* 42 days after hatching, MZUEL 5690, end of postflexion: a) lateral view; b) dorsal view of head; c) left pectoral fin in dorsal view. Scale bar = 1 mm.

Large yolk sac persistent on day 4 (Fig 2a and 2b), much reduced at day 9, and completely consumed within the 13^th^ day of development. In the early stage of postflexion, head deep, with rounded shape in lateral view; depression of head and body starting on day 18. Dorsal region of skull with several ridges on day 19. Mentonian and maxillary barbels larger than in flexion-stage specimens. Outer mentonian barbels and maxillary barbels do not exceed the gill opening. Inner mentonian barbel exceeds posterior margin of eye in early stage of postflexion. Gill opening and nostrils formed. Mouth formed and relatively wide (Figs 3b and 4b); subterminal position persistent until day 17 (Fig 3a), becoming superior after day 27 (Fig 4a). Exogenous feeding is possible after day 3 when the mouth opens.

Primordial fin well developed in early stage of postflexion, surrounding the entire perimeter of trunk and tail; restricted to posterior regions of adipose and anal fins after day 18. Primordial fin further reduced by day 42, but still confluent with caudal fin. Adipose fin defined after 42 days. Dorsal fin distinct in the early stage of postflexion, and fully developed after day 12. Anal fin well developed on day 8, but still associated with primordial fin; final development of anal fin on day 20.

**Fig 5:**
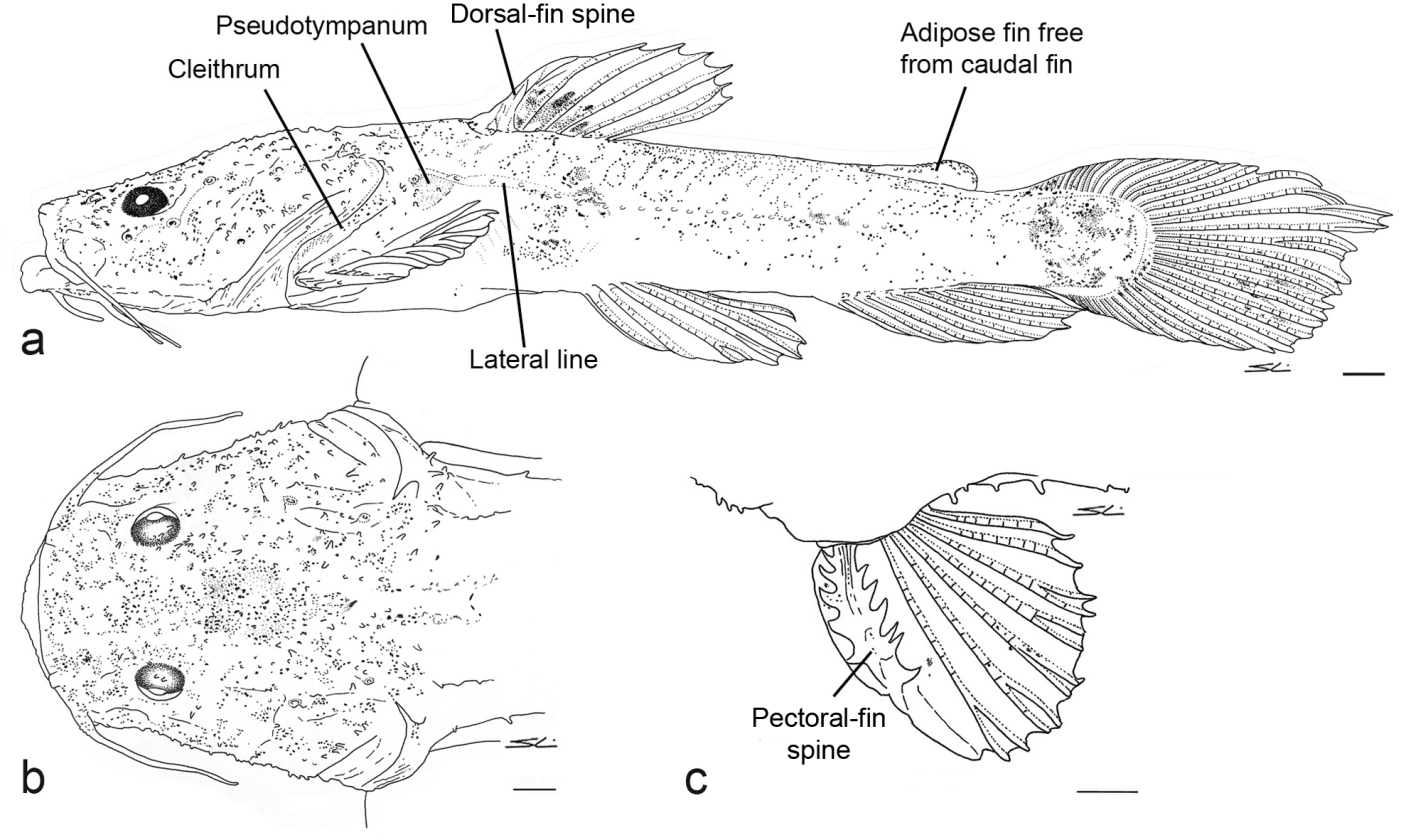
*Lophiosilurus alexandri* juvenile, 60 days after hatching, MZUEL 5694: a) lateral view; b) dorsal view of head; c) dorsal view of left pectoral fin. Small dorsal and ventral procurrent rays not visible. Scale bar = 1 mm.

Pectoral fin formation starting on day 7 with emergence of rays; completely developed after day 15. First ray of pectoral fin begins to differentiate on day 6, with small hooks evident along posterior margin; after day 9, hooks visible on both sides with posterior hooks larger than anterior ones (Fig 4c). At 42 days, hooks on anterior and posterior margins of pectoral-fin spine similar in size (Fig 5c). Cleithral bulge and posterior cleithral process weakly developed on day 6, and well developed by the end of postflexion stage. Spurious ray [20] present at the end of pectoral-fin spine since early in larval development (9 days), and persist in adults. Pelvic-fin bud rounded, base large at the start of postflexion stage (day 4). Rays evident on day 4; well developed on day 10. Incipient caudal fin formed by finfold and confluent with dorsal and ventral portions of finfold; dorsal caudal-fin lobe more developed and slightly longer than ventral lobe, with 15 incipient rays visible in 4-day old specimens (Fig 2a). Distal portion of hypural bones, supporting principal rays of caudal fin [21, 22], emerge as cartilage on day 3 (flexion stage), and begin to ossify on day 13.

In the early stage of postflexion, dark dendritic chromatophores more concentrated on head, dorsal and ventral regions of trunk from nuchal region to tail, and dorsal region of yolk sac (Fig 2a and 2b). Eyes pigmented. In the late stage of postflexion, chromatophores appear more widespread on body, and more concentrated on head, caudal peduncle, and dorsal-fin base (Fig 4a and 4b).

On day 17 (Fig 3a, 3b and 3c), head longer than wide. Dorsal fin well developed, positioned distinctly in front of middle of body. Axillary gland visible above base of pectoral fin. Pectoral fin, when adpressed, reaching vertical through end of dorsal-fin base and slightly surpassing origin of pelvic fin. Pelvic fin, when adpressed, reaching origin of anal-fin base. Anal fin fully formed, last ray separated from ventral finfold. Primordial adipose fin persistent as part of dorsal portion of finfold, confluent with caudal fin; terminus of adipose fin evident as small notch, otherwise confluent with caudal fin. Caudal fin dorsally confluent with adipose fin; ventral finfold with reduced depth, but connected to anal and caudal fins. Caudal fin with seven dorsal procurrent rays, 13 principal rays and seven ventral procurrent rays (Fig 3a). Dorsal lobe of caudal fin slightly longer than ventral lobe. All fin rays segmented.

At the end of postflexion (42 days; Fig 4a, 4b and 4c), head width increases, equals length. Skin of head with small wart-like appendages mainly on supraorbital and ventral regions. Dorsal fin remains positioned anterior to middle of body, distant from head by about half the head length. Pectoral fin, when adpressed, reaching vertical through base of fourth ray of dorsal fin, but not reaching pelvic fin. Origin of pelvic fin at vertical through end of dorsal-fin base; pelvic fin reaching anal fin when adpressed. Adipose fin completely separate from dorsal fin, but with small tissue connection to caudal fin. Anal-fin base shorter than adipose-fin base. Dorsal lobe of caudal fin long, almost twice as long as ventral lobe. Caudal fin with 14 dorsal procurrent rays, 13 principal rays, and 12 ventral procurrent rays (Fig 4a).

#### Juvenile period

**Fig 6:**
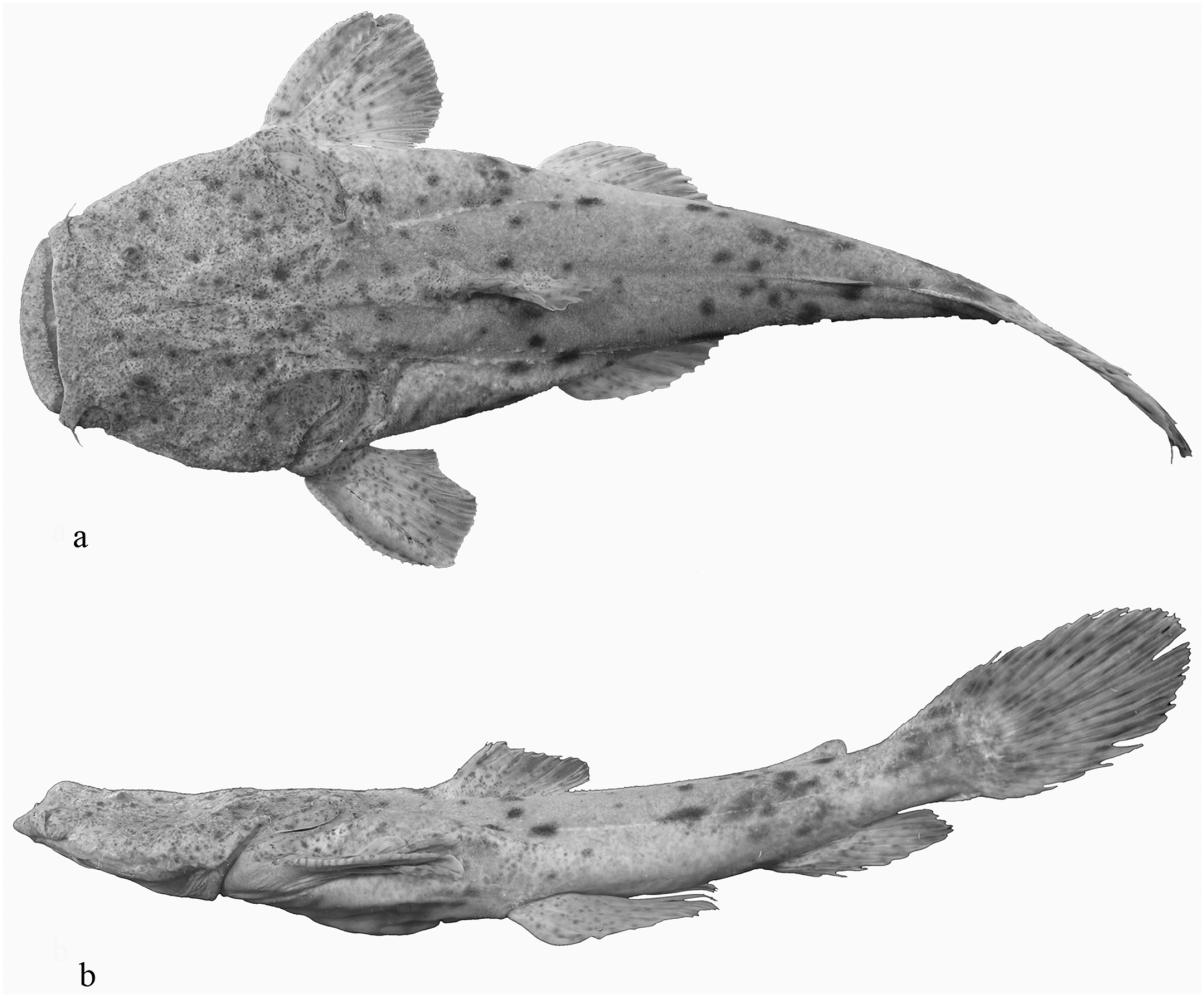
*Lophiosilurus alexandri* adult, MZUEL 1780, 167,17 mm SL: a) dorsal view; b) lateral view.

Stage observed from 49 to 60 days after hatching. Standard length 15.9 to 28.1 mm. Eyes small relative to head (Table 1). Body and head distinctly depressed (Fig 5a); head approximates hexagonal shape in dorsal view (Fig 5b), wider than long; area of frontal bone depressed, concave in coronal section with conspicuous bony ridges. Mouth large, with prognathic position (Fig 5a) similar to adult specimens (Fig 6a and 6b). Maxillary barbel not reaching gill opening. Tip of outer mentonian barbel surpassing tip of maxillary barbel. Tip of inner mentonian barbel not surpassing posterior margin of eye (Fig 5a). Pectoral girdle well developed, particularly cleithral bulge and posterior cleithral process. Pectoral fin with strong anterior spine and seven soft rays; spine thickened by retrorse hooks on anterior and posterior margins (Fig 5c). Dorsal fin well developed with short, thick, strong anterior spine and six soft rays. Adipose-fin base elongate in specimens at 49, 52 and 56 days post-hatching. Anal fin with 12 unbranched rays. Pelvic fin with 6 rays, third and fourth rays branched in specimens of 52 days or older, fifth and sixth rays branched in specimens of 49 days or older.

Dark dendritic chromatophores widespread on head, most concentrated on area of frontal bone; similar chromatophores concentrated on trunk beneath dorsal fin and on caudal peduncle. Base of dorsal fin with chromatophores concentrated in patches. Adipose fin with chromatophores concentrated along base forming a bar that reaches middle of body. Caudal, pectoral and pelvic fins largely hyaline, with sparse dark chromatophores (Fig 5a).

In 60 day old specimens, head wider than long. Dorsal fin in front of middle of body, when adpressed not reaching adipose fin. Skin of head and body with small appendages most evident as bony ridges. Adipose-fin reduced in size with shorter base, separated from caudal fin. Pectoral fin, when adpressed, reaching vertical through dorsal-fin spine, but not reaching pelvic fin. Pelvic fin, when adpressed, no longer reaching anal fin. Anal-fin base becoming longer than adipose-fin base. Caudal fin free from adipose and anal fins, dorsal lobe remains distinctly longer than ventral lobe. Caudal fin with 20 dorsal procurrent rays, 13 principal rays, and 18 ventral procurrent rays. Lateral-line canal well developed to vertical through terminus of dorsal-fin base; lateral line pores evident to vertical through middle of adipose-fin base. Axillary pore very difficult to visualize in larval specimens; associated gland visible by transparency of skin. Only one pore in each side of the body along the surface of the skin under postcleithral process in adult specimens.

### Morphometric analysis

**Fig 7:**
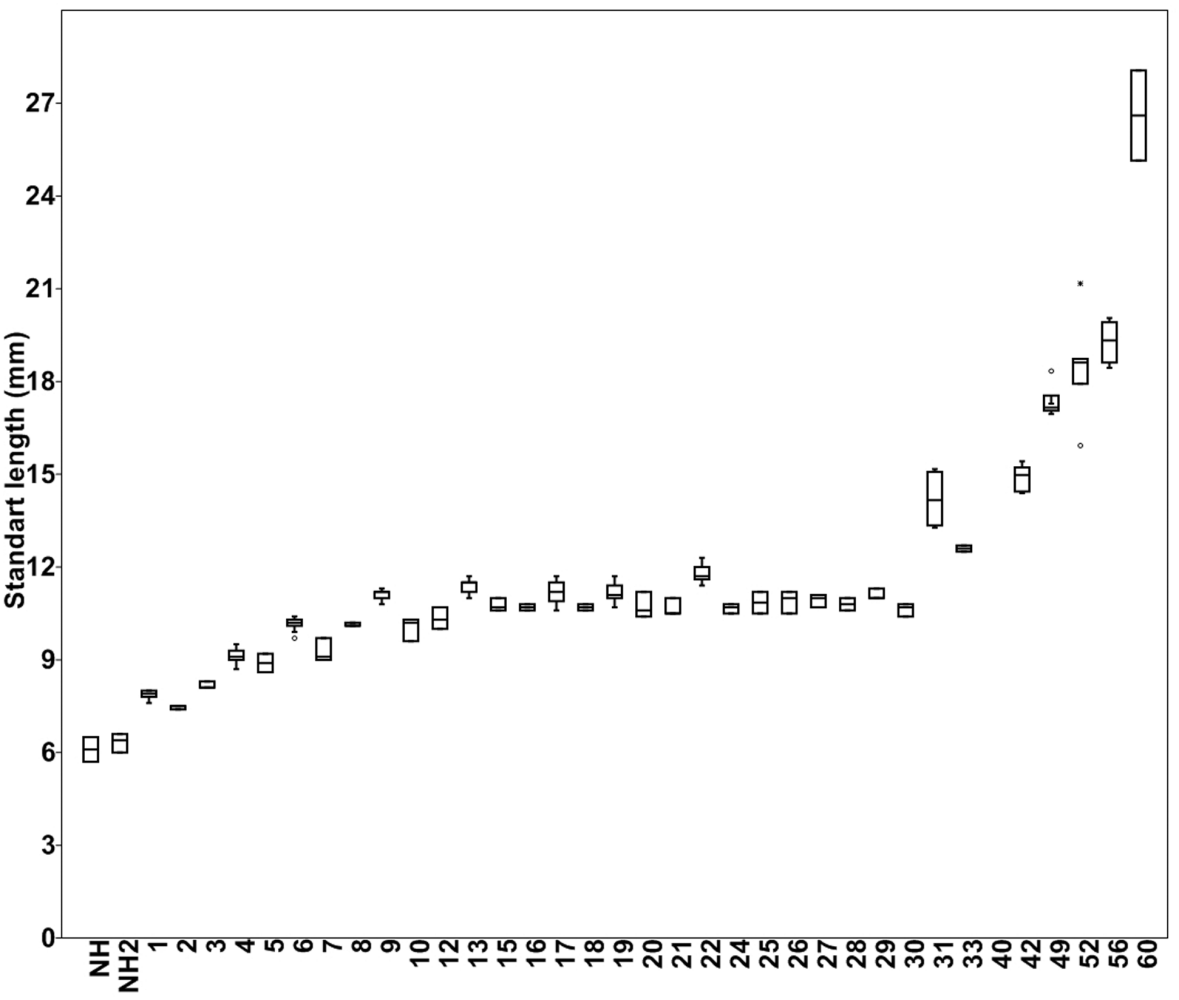
Box plot of standard length by age in days (x-axis) of *Lophiosilurus alexandri*. NH = immediately after hatching; NH2 = newly hatched.

Standard length gradually increasing immediately after hatching to nine days post-hatching; relatively constant from 9 to 30 days post-hatching, then increasing more sharply from 30 to 60 days (Fig 7).

Body proportions that increase from post-larval to juvenile stages are: eye diameter, head length, dorsal-fin base length, preanal length, prepectoral length, prepelvic length, predorsal length, caudal-peduncle depth, body width, interpelvic width, pectoral-fin spine length, dorsal-fin spine length, and pelvic-fin ray length (Table 1). On the other hand, the relative depths of head and body decrease from post-larval to juvenile stages, the same happens to snout length, anal-fin base length, caudal-peduncle length, interorbital width, inner mentonian barbel length, outer mentonian barbel length, and maxillary barbel length (Table 1).

**Fig 8:**
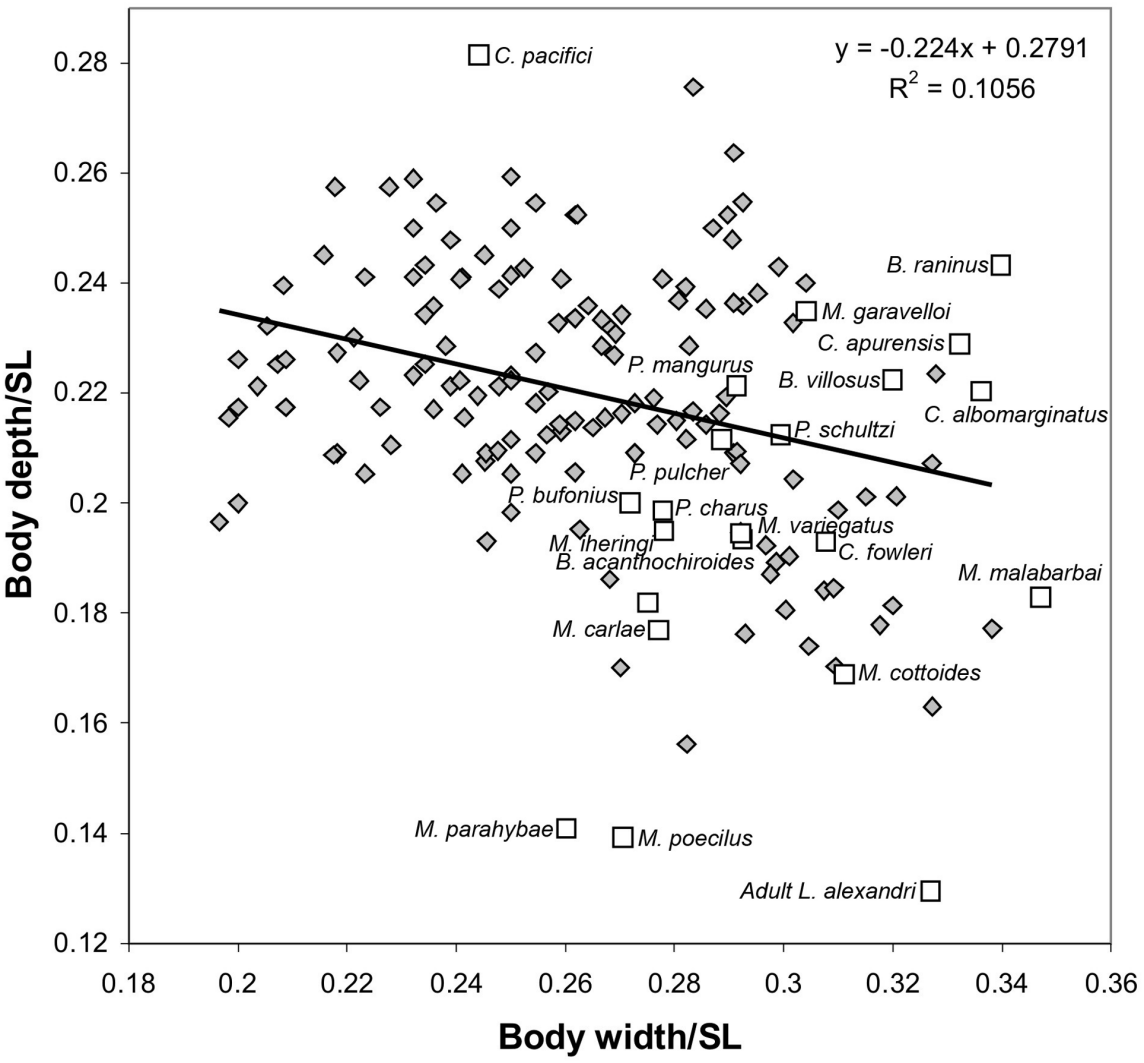
Relation of relative values of body depth to relative value of body width of larvae and juvenile individuals of *Lophiosilurus alexandri* (diamonds) and mean values of other species of Pseudopimelodidae (squares). The regression curve corresponds only to larvae and juveniles of *L*. *alexandri*.

Correlation analysis of body proportions related to body width and depth of larvae and juveniles of *Lophiosilurus alexandri* yielded a large dispersion, but with a tendency of increase the width over depth, as indicated by the regression line (Fig 8). The inclusion of the width and depth mean proportions obtained from juveniles and adults of other species of the family, shows a wide distribution of these species along the X axis (body depth), but with a tendency to a narrower distribution on the Y axis (body width). Even though almost all species fit the variation of depth and width present in larvae and juveniles of *Lophiosilurus alexandri*, three species showed values outside of the aforementioned variation: *Cruciglanis pacifici* has the highest proportion of body depth indicating its relatively deep body, and *Microglanis parahybae* and *M*. *poecilus* have the lowest proportion of body depth indicating its relatively depressed body. The highest value in Fig 8 was obtained by an adult specimen of *Lophiosilurus alexandri*, due to its distinctly depressed body, more depressed than any other species of Pseudopimedidae.

## Discussion

In the development of *Lophiosilurus alexandri*, major transformations in external morphology were observed between larval stages and between larvae and juveniles. In studies with *Hoplias malabaricus* and *Pyrrhulina australis*, respectively, Bialetzki *et al*. [23] and Taguti *et al*. [24] observed similar transformations in external morphology during early development. The present observations also corroborate the Theory of Saltatory Ontogeny [25], since development alternated through periods of slow and small changes and fast and large changes. For example, the plateau in standard length during the postflexion stage in *L*. *alexandri* (i.e., day 9 to 30) may be explained by the allocation of energy resources in the formation of anatomical structures.

Changes in body shape, position of mouth and eyes, and fin morphology were observed during the development of *Lophiosilurus alexandri*. Many of these transitional features are seen as distinct terminal conditions in the other species of Pseudopimelodidae, and have been used as characters in phylogenetic analyses. There are some competing hypotheses of the evolution of Pseudopimelodidae based on the morphological characters proposed by Shibatta [9], Ortega-Lara & Lehman [11] and Birindelli & Shibatta [10]. The interpretation of some of these characters based on their ontogeny instead of their terminal condition in fully developed specimens may add clues to the investigation of the pseudopimelodid evolution.

The relative width and depth of body present significant changes during the development of *Lophiosilurus alexandri*. In fully developed specimens of *Lophiosilurus*, the body is extremely wide and depressed [26], a condition that is unique among pseudopimelodids and therefore autapomorphic. The extreme condition in *L*. *alexandri* is related to its psammophilous and piscivorous behaviors. *Lophiosilurus* is a bottom-up predator that lurks just below the substrate, leaving only the eyes and mouth exposed as it awaits its prey. The highly depressed body shape is also convergent in some well-known species of benthic fish marine such as *Lophius* spp. (Actinopterygii, Lophiidae) and *Squatina* spp. (Chondrichthyes, Squatinidae). However, the condition in larvae and juveniles of *Lophiosilurus* is similar to the one present in the other species of pseudopimelodids. Therefore, the relative depth of the body is a phylogenetic character with at least two states (perhaps more, if a more accurate morphometric analysis is performed), a relatively deep body being plesiomorphic and a relatively depressed body being apomorphic. This interpretation favors the hypothesis that *Cruciglanis* and *Pseudopimelodus* are sister to all other Pseudopimelodidae, both taxa having a relatively deep body (compared to other species of the family). The more shallow bodied *Cephalosilurus* and *Lophiosilurus* are thereby derived and nested within the family.

The position of the mouth has also undergone major changes during the development of *Lophiosilurus*. The mouth is subterminal at the beginning of the yolk sac larval stage. However, the mouth shifts to a terminal position during the end of the yolk sac and early postflexion stages, and eventually becomes superior, prognathous by the end of the postflexion stage, a condition retained into adulthood. This developmental change can also be interpreted as an ordered series of character states. Among adult pseudopimelodids, the putative plesiomorphic state, the subterminal mouth, is present only in *Cruciglanis pacifici*. An intermediate state, the terminal mouth, is present in adult *Batrochoglanis* and some *Microglanis*, sister genera according to Shibatta [9] and Ortega-Lara & Lehmann [11]. A third state, mouth slightly superior, is present in adult *Pseudopimelodus* and some *Microglanis*. The most apomorphic state, mouth superior and prognathous, is present only in adult *Lophiosilurus* and *Cephalosilurus*, corroborating the phylogenetic hypothesis that these two genera form a clade nested within Pseudopimelodidae [9, 11, 10]. Ortega-Lara & Lehmann [11] and Birindelli & Shibatta [10] placed *Lophiosilurus* plus *Cephalosilurus* as sister to all other Pseudopimelodidae.

Eyes placed laterally on the head are a condition shared by all Pseudopimelodidae except for juvenile and adult *Lophiosilurus*. Placement of the eyes on the dorsal surface of the head is a condition unique to juvenile and adult *Lophiosilurus*, and therefore an autapomorphy of the species.

The maxillary barbel is relatively short in *Lophiosilurus*, falling shy of the dorsalmost portion of the gill opening in specimens of all ages. In fact, during the development, the length of the maxillary barbel decreases compared to the length of head, showing negative allometric growth. The maxillary barbel is also short in all other species of Pseudopimelodidae. Actually, the short maxillary barbel resulting from negative allometric growth seems to be a synapomorphy for the entire family, since long maxillary barbels are present in Heptapteridae and Pimelodidae, the two catfish families most closely related to Pseudopimelodidae [27]. Conversely, the maxillary barbel in Heptapteridae and Pimelodidae show positive allometric growth [14].

The caudal fin in *Lophiosilurus* is formed and replaces part of the finfold during the postflexion stage. Initially the dorsalmost rays of the caudal-fin upper lobe are longest than the other rays. However, as specimens grow, the dorsalmost rays of the upper lobe become relatively shorter, and the middle rays of the upper lobe are relatively longer, including the adults. The dorsalmost rays of the upper caudal-fin lobe are also longest in larvae of *Batrochoglanis raninus* [28], indicating that this condition may be also present in the remaining Pseudopimelodidae. Unlike *Lophiosilurus*, as specimens of *Batrochoglanis raninus* grow, the upper lobe becomes similar in size to the lower lobe. Based on this ontogenetic series it is possible to interpret this character (shape of the caudal fin) as having three distinct states. In the plesiomorphic state, the dorsalmost rays of the caudal fin are elongate. In the first apomorphic state, the upper and lower caudal-fin rays are approximately similar in length, and the second apomorphic state, the middle rays of the upper caudal-fin lobe become elongated. There is no indication that this character represents a direct (i.e., ordered) transformation series, because the three states are not present in the developmental series of any species. The most plesiomorphic condition is possibly present in the larvae of all species of Pseudopimelodidae, and in fully developed specimens of *Cephalosilurus albomarginatus* and *Microglanis iheringi*. The presence of this plesiomorphic state in the latter two species can be interpreted as two independent events of paedomorphosis. On the other hand, the condition of elongate middle rays of the upper caudal-fin lobe is only present in *Lophiosilurus*, and therefore, another autapomorphy of the species. The caudal fin is responsible for swimming in most fishes [29], and the upper lobe is distinctly wider than the lower lobe in species that are typically benthic and need fast bottom-up movements [30, 31, 32]. Therefore, the shape of the caudal fin in *Lophiosilurus* is possibly related with its habits as a psammophilous bottom-up predator. The change from the first to the second apomorphic state of this character is also observed in developmental series of many species of Auchenipteridae, Heptapteridae, and Pimelodidae [14, 28], indicating that this may be a evolutionary change that occurred early in the evolution of catfishes.

The pectoral-fin spine of fully developed specimens of *Lophiosilurus* has strong serrations of similar size on the anterior and posterior faces. These serrations are absent in yolk sac and flexion stage larvae, and began to develop in larvae of postflexion stage. The serrations are initially much larger on the posterior face of the spine (Fig 3C), and eventually become similar in size (Fig 4C). Strong serrations of similar size on the anterior and posterior faces of the pectoral-fin spine are present in most species of Pseudopimelodidae. The only exceptions are *Pseudopimelodus* and *Cruciglanis*, which have serrations much larger on the posterior face of the spine. The presence of this plesiomorphic state in the latter two genera can be interpreted as an event of paedomorphosis, corroborating Birindelli & Shibatta [10] for considering them as sister taxa.

The lateral-line system is well developed in adults of *Lophiosilurus*, distributed over the head and as a midlateral canal over the body from head to caudal-fin base. The cephalic portion of the system is formed in the postflexion larvae. However, the lateral-line canal of the body starts to develop in young juveniles from head to rear; the lateral-line canal extends to the vertical through the terminus of the dorsal-fin base in 60-day old specimens. Species of most genera of Pseudopimelodidae also present a well-developed lateral line that extends on the body from head to caudal-fin base. However, some species of the family have an incomplete lateral line wherein the lateral-line canal does not reach the caudal-fin base. This is the case in *Microglanis* and *Batrochoglanis*, wherein the lateral-line canal extends from the head to below the origin of the dorsal-fin base [9], or to the terminus of the adipose-fin base [33], respectively. The presence of an incomplete lateral line on the body is most likely due to a paedomorphic event, and is a putative synapomorphy for a clade composed of *Batrochoglanis* and *Microglanis*, corroborating previous hypotheses [9, 11].

The skin of fully developed specimens of *Lophiosilurus* possesses many finger-like papillae which begin to develop as wart-like appendages in the postflexion larvae. These papillae are formed by an accumulation of keratin, and are distinct from the tubercles present in other catfishes [34, 35, 36, 37] by lacking unculiferous cells [38]. The finger-like papillae probably function as camouflage for specimens of *Lophiosilurus* as they bury in the sand. Similar papillae are absent in most species of the family. Nevertheless, *Cephalosilurus apurensis* also has some papillae on the skin, indicating that it might be possibly closely related to *Lophiosilurus*, corroborating all three hypothesis of evolution of Pseudopimelodidae [9, 11, 10].

Species of most genera of Pseudopimelodidae, including *Lophiosilurus*, have an axillary gland that produces mucus and is located immediately beneath the anterior portion of the posterior cleithral process. In *Lophiosilurus*, this gland starts to develop in flexion stage larvae as a white tissue cluster immediately above the primary pectoral fin. However, in *Batrochoglanis* and *Microglanis*, the axillary gland is absent in adults, a synapomorphy for a clade uniting those genera [9, 11], most likely due to a paedomorphic event.

The coloration of adult specimens of *Lophiosilurus* is greatly marked by the presence of widespread dark dendritic chromatophores, forming an overall dusky ground color. These dark chromatophores, although already present in yolk sac larvae, are only abundant in postflexion larvae, juveniles and adults. According to Nakatani *et al*. [39], most fishes have pelagic larvae that are usually scarcely pigmented, but undergo changes in pigmentation during the juvenile period when the fishes begin to explore different environments and need camouflage. However, as larvae of *Lophiosilurus* are benthic and guarded by the male in excavated nests [40, 12], the change of coloration that takes place in the postflexion stage can be possibly coincident with the onset of parental care. The color pattern of most species of Pseudopimelodidae is formed by dark transverse bars on the body that is somewhat triangular, wide dorsally and tapered ventrally, and dark irregular blotches on the sides of head and body. The only exceptions are *Lophiosilurus*, as described above, and *Cephalosilurus fowleri*. Even though fully developed specimens of the latter have the coloration of *Lophiosilurus*, juveniles of *C*. *fowleri* have the color pattern typical of most species of Pseudopimelodidae. Therefore, the absence of dark bands and blotches in *C*. *fowleri* and *L*. *alexandri* can be interpreted as independently acquired, both conditions being autapomorphic.

The analysis presented herein shows that the study of the development of *Lophiosilurus alexandri* helps to determine the limits and polarity of the character states related to the evolution of the Pseudopimelodidae, especially for sister taxa relationships, such as those of *Batrachoglanis* and *Microglanis*, *Lophiosilurus* and *Cephalosilurus*, and *Cruciglanis* and *Pseudopimelodus*, conditions observed in larval and juvenile stages of *L*. *alexandri* illuminate how character states are connected, and their homologies across taxa in the family. Noteworthy to say that some of the features thought to be completely modified in adults of *Lophiosilurus alexandri* (autapomorphic), and therefore uninformative for the evolution of other species of Pseudopimelodidae, were in fact part of a complex character-state series that is traceable through ontogeny.
